# An IoT Reader for Wireless Passive Electromagnetic Sensors

**DOI:** 10.3390/s17040693

**Published:** 2017-03-28

**Authors:** Gabriel Galindo-Romera, Javier Carnerero-Cano, José Juan Martínez-Martínez, Francisco Javier Herraiz-Martínez

**Affiliations:** Department of Signal Theory and Communications, Universidad Carlos III de Madrid, Av. de la Universidad 30, 28911 Leganés, Madrid, Spain; jccano@tsc.uc3m.es (J.C.-C.); jmartinez@tsc.uc3m.es (J.J.M.-M.); fjherraiz@tsc.uc3m.es (F.J.H.-M.)

**Keywords:** Internet of Things, reader, passive electromagnetic sensor, wireless link, laboratory measurements, split-ring resonator (SRR), permittivity measurements, low-cost sensing system, portable sensing system

## Abstract

In the last years, many passive electromagnetic sensors have been reported. Some of these sensors are used for measuring harmful substances. Moreover, the response of these sensors is usually obtained with laboratory equipment. This approach highly increases the total cost and complexity of the sensing system. In this work, a novel low-cost and portable Internet-of-Things (IoT) reader for passive wireless electromagnetic sensors is proposed. The reader is used to interrogate the sensors within a short-range wireless link avoiding the direct contact with the substances under test. The IoT functionalities of the reader allows remote sensing from computers and handheld devices. For that purpose, the proposed design is based on four functional layers: the radiating layer, the RF interface, the IoT mini-computer and the power unit. In this paper a demonstrator of the proposed reader is designed and manufactured. The demonstrator shows, through the remote measurement of different substances, that the proposed system can estimate the dielectric permittivity. It has been demonstrated that a linear approximation with a small error can be extracted from the reader measurements. It is remarkable that the proposed reader can be used with other type of electromagnetic sensors, which transduce the magnitude variations in the frequency domain.

## 1. Introduction

In the last few years, many electromagnetic, microwave or radiofrequency (RF) sensors have been reported in the literature (some good examples are [1,2,3,4,5,6]). One important advantage of electromagnetic or RF sensors is that they can be easily integrated with antennas, resulting in wireless devices (e.g., [5,6]). One of the current trends in electromagnetic sensors is the use of radiofrequency identification (RFID) approaches. A typical RFID system consists of two elements: the reader and the tags [7]. Each tag contains a unique identification code. The reader interrogates the tags located within the read range, and the tags reply to the reader with their identification codes. In order to follow this operation protocol, most tags contain two elements: an antenna and a RFID chip. During the last decade wireless sensors based on RFID chips have been proposed [8,9,10,11,12,13]. However, the inclusion of RFID chips increases the price of tags and sensors, limiting its success. For this reason, chipless passive technologies (RFID tags and sensors) have been proposed [14,15,16,17,18]. In spite of its drawbacks, for instance, the shorter range and the more limited number of bits or information than in chip-based RFID sensors, chipless sensors are a simpler and cheaper alternative for passive sensing and the best solution for disposable sensors.

Most of the previous works focus only on the sensor part, while ignoring the reader. This philosophy leads to the use of general purpose, complex and highly expensive laboratory equipment which can be only operated by trained personnel to read the sensor response. For this reason, the development of specific reading systems for electromagnetic sensors is crucial for the success of this technology.

Electromagnetic chipless sensors can be designed as fully passive wireless devices. These characteristics make this kind of sensors a good candidate for the Internet of Things (IoT). The IoT is the connection of physical items to communication networks (e.g., the Internet) that enable these items to collect and exchange data [19]. This global infrastructure represents the next step towards the digitization of our society and economy [19,20,21,22,23]. The development of sensor systems with networking and cloud capabilities is crucial for the final establishment of the IoT [20,21,22,23]. Some recent proposals on sensors, interfaces and platforms for the IoT can be found in the literature [24,25,26,27,28,29]. These works show potential applications of the IoT on different areas, such as industrial processes [24,25], home monitoring [26] or health and telemedicine [27,28,29]. However, up to the authors’ knowledge, there are no IoT platforms for reading the response of passive wireless electromagnetic sensors.

In this work, a novel IoT reader (IoT-R) for passive wireless electromagnetic sensors is proposed. This reader scheme can be used to interrogate passive electromagnetic sensors within a short-range wireless link. For example, this is useful for the remote monitoring of harmful substances. The proposed IoT-R is based on an innovative layer scheme which is firstly presented in this article. The implementation of this proposal results on a portable, easy-to-operate and low-cost solution. Moreover, the addition of IoT functionalities to the reader allows remote sensing from computers or handheld devices. Other applications such as automatic cloud storage or cloud computing (e.g., cloud postprocessing of the data sent by the reader) are possible with the proposed technology. The remote operation of the IoT-R is fully automated and efficient, reducing the human errors in acquisition, processing and storage of sensing data. Finally, a full implementation of an IoT-R for wireless sensors is presented in this article. The objective is to show a fully functional demonstrator of the proposed technology. This demonstrator is devoted to the remote sensing of the dielectric permittivity of liquids, but the same approach can be applied to sense other magnitudes by changing the passive wireless sensors. A full IoT-R implementation for the proposed application is designed and manufactured. Moreover, the remote sensing of different liquids is performed, obtaining an accurate measurement of the dielectric permittivity.

The development of the proposed demonstrator has been very challenging because several technologies have been involved in an efficient manner to fulfil the objetives of the work achieving a low-cost IoT-R. First, the proposed sensing system and layer-scheme-based IoT-R (Section 2.1) are major contributions of the work because these paradigms can be applied to any passive wireless electromagnetic sensor system, resulting in low-cost but fully functional applications. In this paper, the submersible sensor presented in [30] has been chosen to show the validity of the proposed schemes. However, in the previous work the sensor was directly wired to complex and expensive laboratory instruments. In the present work, the sensor has been connected to antennas and the IoT-R is capable of detecting the backscattered signal from the sensor, which depends on the substance under measurement (Section 2.2). Moreover, a specific RF interface is designed to interrogate the sensor (Section 2.3). All the different circuits which compose this RF interface have been optimized for the application, achieving a low-cost and compact solution. Then, the detected signal is digitized and trasmitted through the Internet. In order to implement these functions a low-cost mini-computer has been used. Moreover, a specific protocol has been designed and implemented. The proposed communication protocol has been mounted over the TCP-IP stack to achieve remote sensing through the Internet (Section 2.4). Finally, a method to obtain the dielectric permittivity from the signals sent through the Internet is proposed in Section 3. This approach is more elaborated that the direct measurement with laboratory instruments that it is usually performed (e.g., [30]), but the resulting mean error is very low.

## 2. Materials and Methods

### 2.1. Sensing System and IoT Reader Definitions

**Figure 1:**
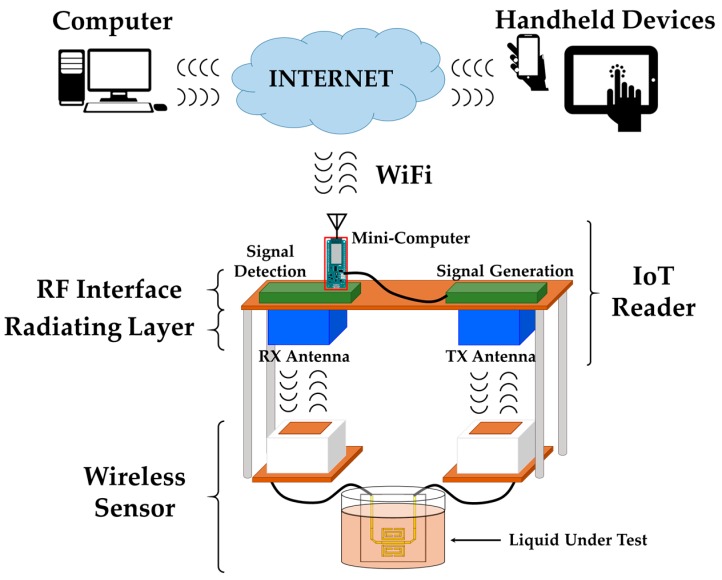
Schematic of the proposed IoT sensing system.

The general sketch of the sensing system is shown in Figure 1. It is based on two elements: a passive wireless electromagnetic sensor and an IoT-R. The sensor is capable of backscattering a wireless electromagnetic signal which depends on the magnitude under measurement. The sensor is interchangeable. Thus, the proposed system can be used to remotely sense different magnitudes by changing the sensor. Moreover, the sensor can be easily replaced in case of failure or damage. In the present work, the system will be used to obtain the dielectric permittivity of different liquids by immersing the sensor in each of the liquid under test (LUT). The sensor is based on printed metamaterials particles without additional electronics, leading to a low-cost, replaceable, battery-free and fully passive wireless solution. The description of the sensor used in this work is provided below. The system also contains an IoT-R, which generates the radiofrequency signal to interrogate a wireless sensor, receives the sensor respones, digitizes that signal and acts as the IoT interface of the laboratory. The presence of the IoT interface implies that the control of the system is made remotely by a computer or handheld device (e.g., smartphone or tablet) connected to the reader through the Internet. Thus, the working principle of the proposed system is as follows: the laboratory technician inmerse the sensor in a LUT; then, the reader takes a wireless measurement from the sensor when it receives a sensing request from a client through the Internet. Finally, the IoT-R sends the result of the measurement back to the client through the Internet. This operation protocol is valid for the characterization of different LUTs, but it is possible to remotely monitor the changes of the same LUT (e.g., changes with time or temperature) by maintaining the sensor immersed in the same LUT and remotely sending periodic requests to the reader.

The final objective of this article is the development of a functional demonstrator including the passive wireless sensors, the IoT-R and remote control and acquisition functions through the Internet. In order to show the validity of the demonstrator, it is devoted to a particular application: the characterization of the relative permittivity of different liquids. It is important to note that the proposed paradigm may be applied to the detection of other physical magnitudes by changing the sensors, as commented before.

In order to implement the proposed application, a submersible electromagnetic printed sensor presented by the authors in [30] is used. The sensor is based on a pair of printed rectangular split-ring resonators (SRRs) coupled to a microstrip line. The SRRs introduce a notch in the transmission coefficient ($S_{21_{sens}}$) between the ports of the microstrip line. The sensing principle relies on the frequency shift of the notch when the SRRs are submersed into different LUTs. In [30] it has been demonstrated that the sensor nocth varies between 1.6 and 1.9 GHz, corresponding to a relative permittivity range between 1 and 9. In this work it has been tested with permittivities between 1 and 6, due to the impossibility to find non-polar liquids with relative permittivities out of that range. In order to make a wireless system, the sensor ports are connected to two patch antennas which are presented in Section 2.2. The authors are working on the integration of the sensor and its antennas, but this topic is out of the scope of the present article.

**Figure 2:**
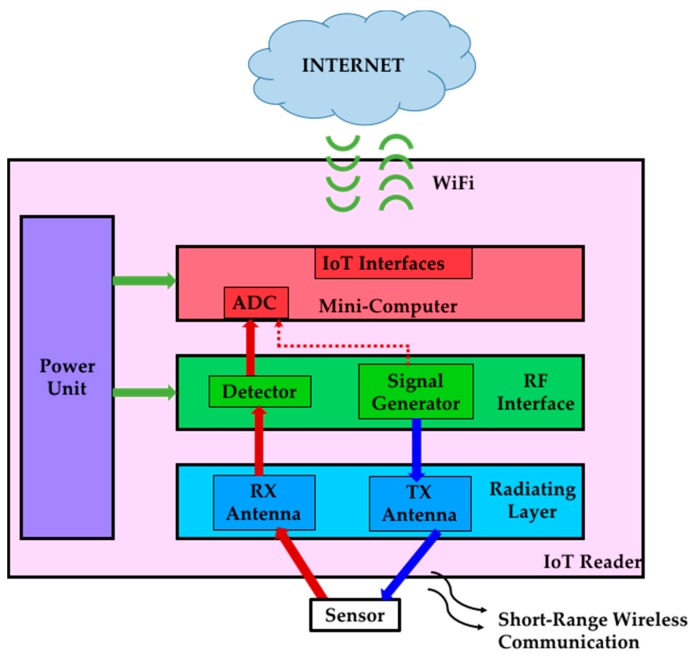
Layer diagram of the IoT reader.

The IoT-R follows the diagram of Figure 2. It is based on four functional layers: the radiating layer, the RF interface, the IoT mini-computer and the power unit. The functions of the RF interface are the generation of a periodic frequency sweep to interrogate the sensor and the detection of the signal modified by the sensor. The radiating layer is an antenna system which wirelessly transmits the signals generated by the RF interface and receives the modified signal backscattered by the sensor. This received signal is transferred to the RF detector, which is a part of the RF interface. The analog signals generated by the detector are sent to the IoT mini-computer. This element digitizes the measurement and transmits it to a client through the Internet. In order to do that, the mini-computer has analog inputs with analog-to-digital (ADC) converters and the IoT control logic and interface (e.g., WiFi) to communicate through the Internet with remote clients. There are low-cost and low-power commercial IoT mini-computers that can be used for this application (e.g., Arduino MKR1000 [31] which includes the ADCs and IoT functions). All the active elements of the different layers are powered by the power unit. The low-cost and small physical dimensions of all the components make the IoT-R a portable device with reduced cost. A full implementation of the IoT-R is presented in this paper. The different layers of the proposed IoT-R implementation are presented in the following Section 2.2, Section 2.3 and Section 2.4. Finally, this work is completed with a software client (programmed in MATLAB [32]) to remotely interrogate the IoT-R when the wireless sensor is immersed in different LUTs. The sensing results of the whole system are shown in Section 3.

### 2.2. Radiating Layer

**Figure 3:**
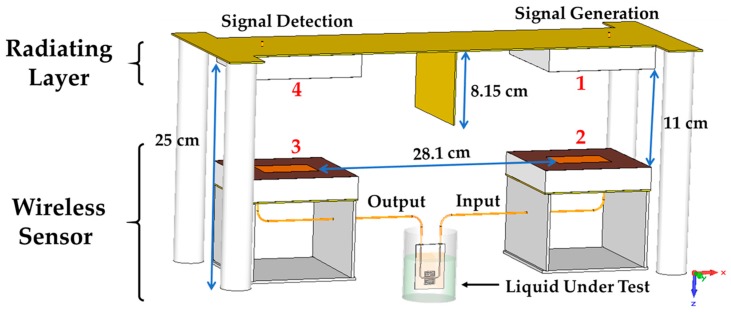
Bistatic system schematic.

Regarding the radiating layer, it is fundamentally inspired on a bistatic system. The radiating layer schematic is shown in Figure 3. In the proposed scheme, four broadband patch antennas are used: two antennas for the reader (transmission and reception) pointed to two antennas connected to the sensor ports. Furthermore, a coupling level between the reader antennas lower than −30 dB can be considered suitable for this application in order to reduce unwanted interferences between the signal generation and detection stages of the reader. Thus, a brass wall (8.15-cm height and 1.56-mm thickness) has been placed between the antennas of the reader, in order to increase their isolation. Besides, the reader antennas coupling can be further decreased if we choose an appropriate gap between them. Nevertheless, it is important not to oversize the system, and hence a trade-off gap is selected. We have optimized this gap through the software Computer Simulation Technology (CST) Microwave Studio, and we have obtainded a 28.1-cm gap between the metallic patches of the reader. Finally, we consider a distance of 11 cm between the reader antenna and the tag antenna, since this distance is useful in laboratory environments (short-range wireless communication). Four plastic cylindrical supports hold the reader during the measurement setup.

A wireless line-of-sight (LoS) link is established between the four antennas. For simplicity, we consider two equal antennas for the reader and two equal antennas for the sensor. The gain of the reader and sensor antennas are $G_{a_{r}}$ and $G_{a_{s}}$, respectively. Moreover, the mispointing and polarization losses will be neglected in the present approach, since it is assumed that the antennas are perfectly pointed and have the same polarization. The thermal noise introduced by the channel, the external interferences, and the statistical nature of the detection process are also considered negligible; as well as other propagation effects. On the other hand, we have to take into account that the SRR-based sensor is passive and linear, and it can be characterized by its scattering parameters (${\left[S\right]}_{s}$). Moreover, since it is reciprocal and symmetrical, we can consider that $S_{21_{s}}=S_{12_{s}}$, and $S_{11_{s}}=S_{22_{s}}$.

The operation, according to Figure 3, is as follows: a signal generator is connected to the antenna 1, whose reflexion coefficient is $S_{11_{a_{r}}}$. This signal is wirelessly transmitted from the antenna 1 to the antenna 2, which is connected to the input port of the sensor; then, the signal travels through the sensor, submersed in the LUT, to the output port where the antenna 3 is connected. The power transmission coefficient between the two antenna sensors is $T_{s}$. Finally, the antenna 3 sends back the signal, modified by the sensor, to the antenna 4 and the signal is detected by the signal receiver. Then, the link budget (in power units), ${\left|S_{21_{rad}}\right|}^{2}$, can be deduced from the Friis transmission equation [33] as:

(1) $${\left|{S_{21}}_{rad}\right|}^{2}=T_{s}{\left(\frac{\left(1-{\left|S_{11_{a_{r}}}\right|}^{2}\right)G_{a_{r}}G_{a_{s}}}{{\left(\frac{4\pi r}{c_{0}}f\right)}^{2}}\right)}^{2}$$

where $c_{0}$ is the speed of light in vacuum, f is the working frequency, and r is the distance between the reader and the tag antennas. Equation (1) is valid because the antennas are operating in the far field region at this distance according to [33]. The transducer power gain equation demonstrated in [34] for arbitrary two-port networks, characterized by their scattering matrix [*S*], and with arbitrary source and load impedance, can be particularized for the proposed sensor system. In this scheme, the passive sensor is the two-port network and the two sensor antennas can be modelled as the source and load impedances, both with $S_{11_{a_{s}}}$ reflection coefficient. From the S-parameters of the SRR-based sensor and the sensor antennas reflexion coefficient, $T_{s}$ can be computed as:

(2) $$T_{s}={\left(\left|{S_{21}}_{s}\right|\frac{\left(1-{\left|S_{11_{s}}\right|}^{2}\right)}{\left|{\left(1-S_{11_{s}}S_{11_{a_{s}}}\right)}^{2}-S_{21_{s}}^{2}S_{11_{a_{s}}}^{2}\right|}\right)}^{2}\approx \;{\left|{S_{21}}_{s}\right|}^{2}$$

since the sensor antennas and the two-port SRR-based sensor are designed to be matched to the system reference impedance (50 Ω) within the working bandwidth, as it will be shown in the system implementation. Hence, from Equation (2) and considering that the reader antennas are matched to the reference impedancce as well, Equation (1) reduces to:

(3) $${\left|{S_{21}}_{rad}\right|}^{2}\;\approx \;{\left(\left|{S_{21}}_{s}\right|\frac{G_{a_{r}}G_{a_{S}}c_{0}^{2}}{{\left(4\pi rf\right)}^{2}}\right)}^{2}\;$$

From Equation (3), it can be seen that the transmission coefficient of the whole radiating layer is directly proportional to the transmission coefficient of the SRR-based sensor. Thus, the frequency notch produced in ${\left|S_{21_{s}}\right|}^{2}$ are appreciated as well in ${\left|{S_{21}}_{rad}\right|}^{2}$, allowing the detection of different LUTs.

Regarding the antennas implementation, the same design has been used for the four antennas. They have been designed to cover the whole working bandwidth of the sensing system. Patch antennas have been chosen because they are easy to manufacture and low-cost. Moreover, their radiation characteristics (broadside pattern and gain) are suitable for the application. However, conventional patch antennas are resonant antennas. For this reason, the antennas used in this paper are based on the broadband design proposed in [35]. These antennas are based on a microstrip patch using L-probe feeding system, achieving the whole bandwidth of the system. On the other hand, we must also consider that the mentioned brass wall slightly influences the reader antenna gain, so it will not be exactly the same as the sensor antenna gain.

**Figure 4:**
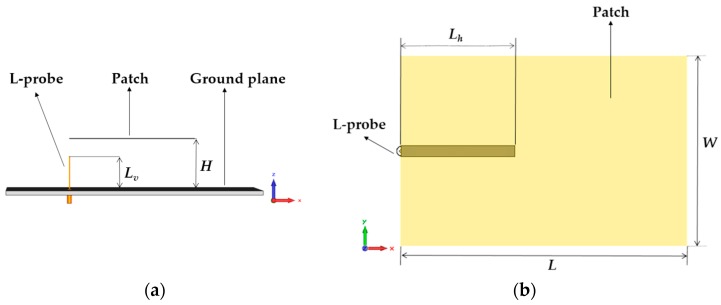
Sketch of the proposed broadband patch antenna. Side view (**a**) and top view (**b**).

In order to cover the desired bandwidth, the following antenna dimensions are determined from [35] (Figure 4): L = 62.5 mm, W = 41.6 mm, L_h_ = 25 mm, L_v_ = 16 mm, and H = 25 mm. Moreover, the antenna substrate is Rohacell 51 HF foam (εr = 1.057 and tan δ = 0.0002), covered by a copper metallized 80-µm thin-film of Kapton. In addition, the ground plane material is brass (1.56-mm thickness).

**Figure 5:**
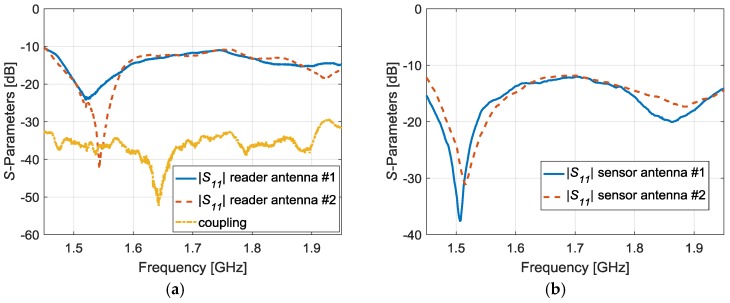
Measured S-Parameters of the manufactured reader antennas (**a**) and the sensor antennas (**b**).

The four antennas have been manufacturated and placed in the measurement setup. Then, the measured reflexion coefficient of the reader antennas (Figure 5a) and sensor antennas (Figure 5b) are represented below. It can be observed that the four antennas achieve the matching criterion ${\left|S_{11}\right|}_{dB}\leq$ −10 dB within the system frequency range. Moreover, a satisfactory isolation level between the antennas of the reader has been accomplished because the coupling is below −30 dB (Figure 5a).

**Figure 6:**
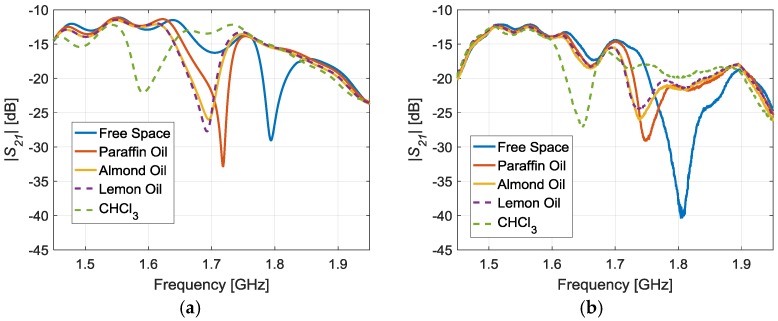
Simulated (CST Microwave Studio) (**a**) and measured (**b**) radiating layer transmission coefficients for the different LUTs.

The final step is the demonstration that the proposed radiating layer can detect different LUTs, even with close values of relative permittivity. Figure 6 shows the simulations, performed in CST Microwave Studio (Transient Solver), and the corresponding measurements of the whole radiating system. The measurements have been taken with the Keysight N9918A Vector Network Analyzer (VNA) [36]. The objective of the simulations is to validate that the trend of the resonant frequencies is consistent to the measurements. There is a good agreement between both cases, although a frequency shift is observed between all the simulated and measured results. It is remarkable that these simulations require a high computational cost. Moreover, the simulator used is not optimum for highly resonant structures (like the SRR-based sensor) which might lead to undesired frequency shifts in the simulations. Moreover, there are additional factors in the measurements, such as interferences and multipath effects, which are neither contemplated in the simulations.

The addition of all the effects causes the frequency shift between the simulations and measurements. Anyway, it is clear that the radiating layer operation is correct, since the resonance frequency shifts according to the value of the relative permittivity of the LUTs.

Table 1 shows the corresponding measured results. The relative permittivity of the LUTs was characterized in [30]. The experimental resonant frequency decreases when increasing the relative permittivity of the LUT, as expected. Differences are detected even in the case of almond and lemon oils, which have close relative permittivity values.

### 2.3. RF Interface

Figure 1 shows the schematic of the proposed IoT sensing system. It can be noticed that the RF interface is divided into two different blocks: the signal generator and the signal detector. The signal generator performs a continuous frequency sweep between 1.6 and 1.9 GHz. On the other hand, the signal detector is based on a RF broadband amplifier and a square-law Schottky detector which produces a low-frequency periodic signal related to the sensor response.

#### 2.3.1. Signal Generator

**Figure 7:**
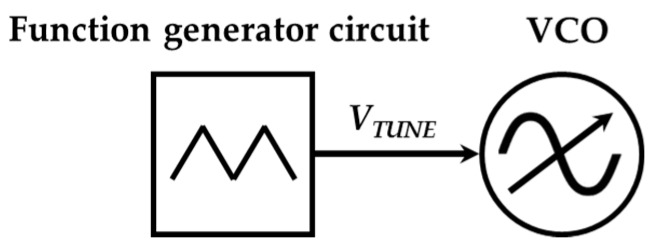
General sketch of the signal generator.

The general sketch of the signal generator is shown in Figure 7. It is mainly based on a voltage controlled oscillator (VCO) whose oscillation frequency is controlled by the input voltage (*V_TUNE_*). The input voltage determines the instantaneous oscillation frequency of the VCO. The input voltage of the VCO is generated by a monolithic function generator integrated circuit. The function generator produces a low-frequency periodic triangular signal which forces the VCO to perform the required frequency sweep. In particular, the VCO generates an increasing sweep and a decreasing sweep of the whole sensor operation band per period. This means that the sensor will response with a periodic double notch (one during the increasing sweep and another during the decreasing one). These notches are associated to the resonant frequency of the sensor immersed in a particular LUT. This will be used to determine the desired sensing magnitude in Section 3.

**Figure 8:**
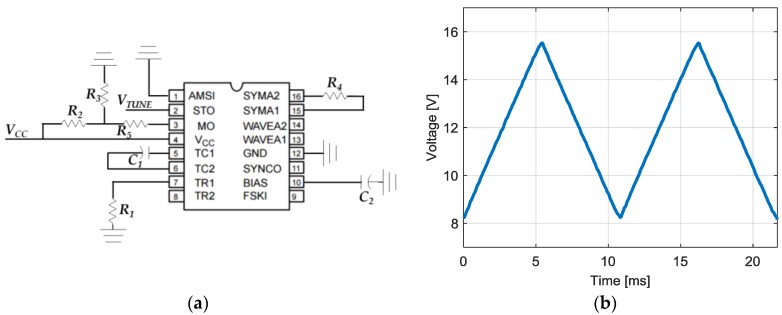
Triangular signal generator configuration (**a**) and its measured triangular signal (**b**).

The chosen VCO is the model JTOS-2000+ manufactured by the company Mini-Circuits (New York, NY, USA). The datasheet [37] specifies that the VCO can be operated between 1.37 and 2 GHz for an input voltage between 6 and 17 V. The output power level of the VCO is 12 dBm approximately. The triangular function generator is based on the Exar Corporation XR-2206 monolithic function generator integrated circuit [38]. The signal generator is configured to generate a triangular signal of period *T* = 10 ms approximately. The used configuration is shown in Figure 8a where *V_CC_* = 24 V, *R*_1_ = 10 kΩ, *R*_2_ = 5.1 kΩ, *R*_3_ = 5.1 kΩ, *R*_4_ = 5.1 kΩ, *R*_5_ = 20 kΩ, *C*_1_ = 1 μF and *C*_2_ = 1 μF. The frequency of the triangular signal is determined by *f* = 1/(*R*_1_·*C*_1_) (≈100 Hz). The DC level at the output (Pin 2, *V_OUT_*) is approximately the same as the DC bias at Pin 3. In this case, the Pin 3 is biased through a voltage divider to give an output DC level of *V_CC_/2*. The amplitude of the output triangular signal is controlled with *R*_5_. *R*_4_ provides a fine adjustment for the waveform symmetry. The measured triangular signal with the proposed configuration is shown in Figure 8b. It has been measured with the DSO-X 3034A digital oscilloscope (Agilent, Santa Rosa, CA, USA) [39]. It can be seen that the voltage varies between 8.25 and 15.50 V while the oscillation frequency is 92.20 Hz. This input voltage signal, *V_TUNE_*, is appropriate to generate the required frequency sweep between 1.6 and 1.9 GHz with the VCO.

**Figure 9:**
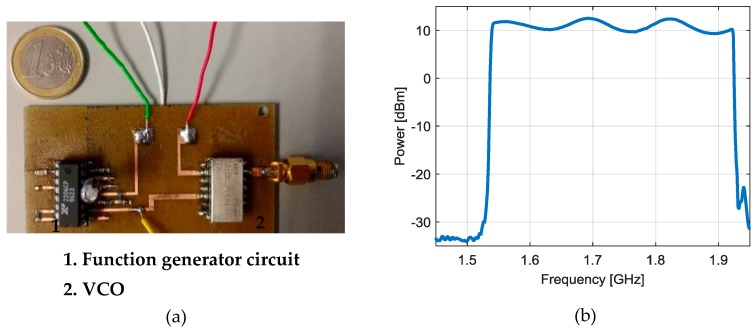
Manufactured signal generator (**a**) and its measured power spectrum (**b**).

Figure 9a shows the picture of the manufactured signal generator, which contains the previous triangular signal generator connected to the VCO. Figure 9b shows the measured power spectrum of the generated signal. The manufactured circuit covers the desired frequency range to interrogate the sensors with an output power over 10 dBm.

#### 2.3.2. Signal Detector

**Figure 10:**
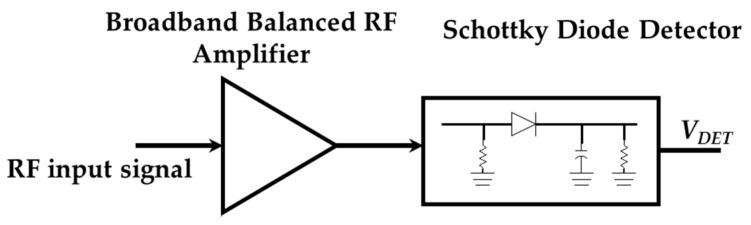
General sketch of the signal detector.

The general sketch of the signal detector is shown in Figure 10. The signal detector is composed by a broadband RF amplifier and a Schottky diode detector (SDD). The RF amplifier mitigates the free space losses between the antennas within the wireless link. This is because it is necessary to achieve a signal detectable by the SDD over −15 dBm. The SDD produces an output voltage proportional to the power of the signal received from the sensor.

**Figure 11:**
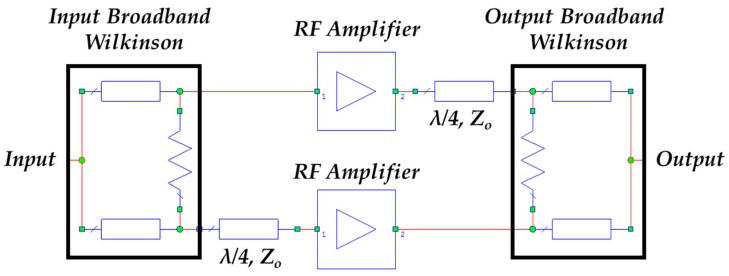
Block diagram of the broadband balanced amplifier.

Figure 11 shows the block diagram of the proposed broadband RF amplifier. It is based on a balanced design [40]. A balanced amplifier typically employs two quadrature couplers and two amplifiers designed in the same way. The quadrature coupler situated at the input of the balanced design produces two signals with equal amplitude and 90° phase-shift at the two amplifiers inputs. Then, the second quadrature coupler at the output of the balanced scheme combines the amplified signals in phase. In the proposed design, a three-stage Wideband Wilkinson Power Divider [34] with an additional 90° phase-shifter in one of its outputs has been used instead of the typical quadrature coupler.

**Figure 12:**
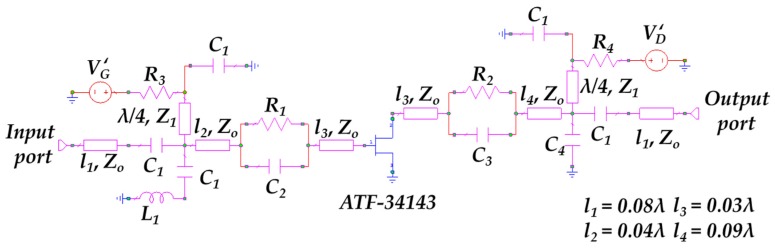
Circuit schematic of the two RF amplifiers.

The broadband amplifier is etched on the commercial dielectric substrate AD1000 (Rogers, Connecticut, CT, USA) with a relative dielectric constant *ε_r_* = 10.6, loss tangent *tan* δ = 0.0023, substrate thickness *h* = 1.27 mm and a metallization thickness of 17 μm. Figure 12 shows the circuit schematic of the amplifiers where *C*_1_ = 1 nF, *C*_2_ = 1.8 pF, *C*_3_ = 3.3 pF, *C*_4_ = 1.8 pF, *L*_1_ = 2.2 nH, *R*_1_ = 150 Ω, *R*_2_ = 82 Ω, *R*_3_ = 1 Ω and *R*_4_ = 100 Ω. The amplifiers are based on the heterostructure field-effect transistor (HFET) model Broadcom ATF-34143 [41]. The characteristic impedance of the high-impedance DC-blocking lines is *Z*_1_ = 95 Ω while for the other lines is *Z_o_* = 50 Ω. The input and output matching networks are based on a resistive model to achieve a flat response in terms of gain (*|S*_21_*|*) and isolation (*|S*_12_*|*) within the whole broad bandwidth.

**Figure 13:**
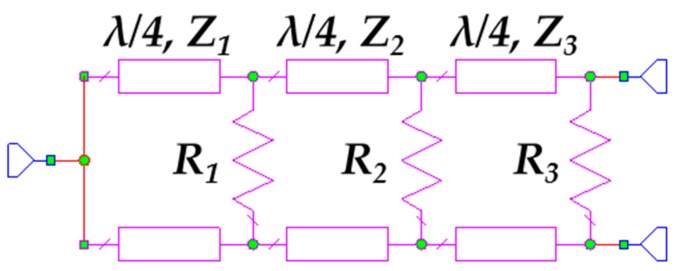
Schematic of the three-stage wideband Wilkinson power divider.

Figure 13 shows the design of the wideband Wilkinson power divider where *R*_1_ = 120 Ω, *R*_2_ = 220 Ω and *R*_3_ = 270 Ω and all the transmission lines have a length of λ/4. The characteristic impedances of the microstrip lines are *Z*_1_ = 90 Ω, *Z*_2_ = 70 Ω and *Z*_3_ = 55 Ω.

**Figure 14:**
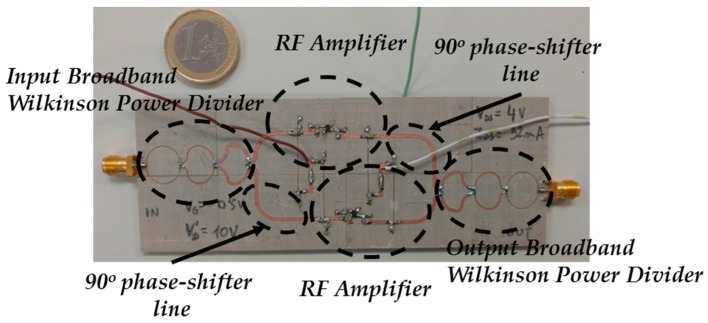
Manufactured balanced RF amplifier.

**Figure 15:**
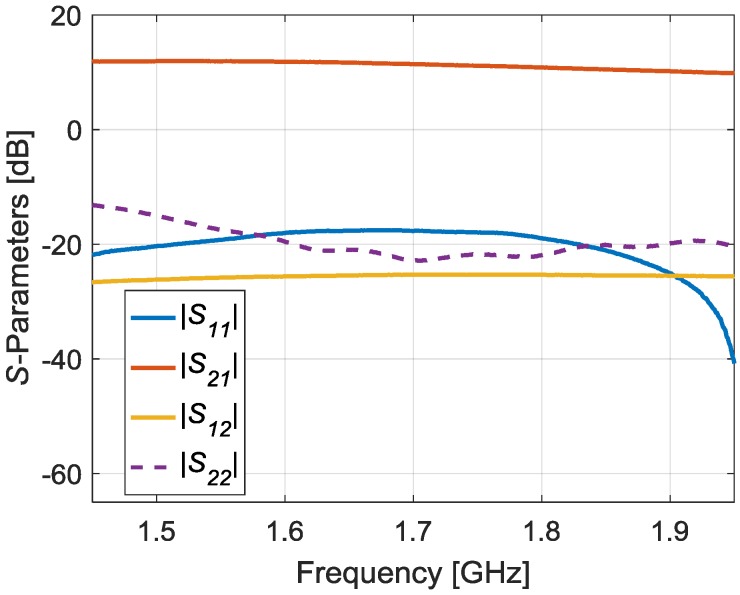
Measured S-Parameters of the manufactured broadband balanced RF amplifier.

Figure 14 shows a picture of the manufactured balanced RF amplifier and Figure 15 shows its measured S-Parameters. It can be seen a good performance over the whole required frequency bandwidth (between 1.6 and 1.9 GHz). The reflection coefficient at the input port (*|S*_11_*|*) is below −17.94 dB while at the output port (*|S*_22_*|*) is below −19.54 dB within the bandwidth of interest. The gain (*|S*_21_*|*) is 11.84 dB at 1.6 GHz and decreases up to 10.20 dB at 1.9 GHz. Finally, a good level of isolation is achieved, presenting the *|S*_12_*|* parameter a value below −25.31 dB.

**Figure 16:**
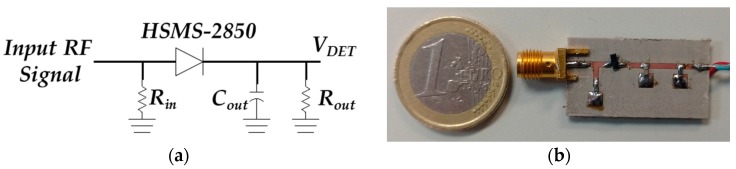
Schematic (**a**) and manufactured SDD (**b**).

**Figure 17:**
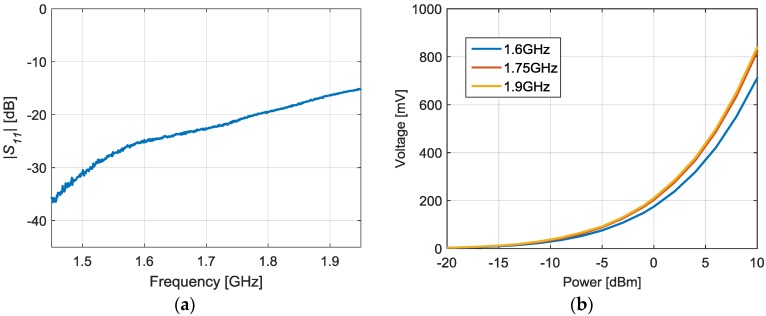
Characterization of the proposed SDD. Measured reflection coefficient (|S_11_|) (**a**) and detected voltage versus the input power for 1.6, 1.75 and 1.9 GHz (**b**).

Figure 16 shows the proposed SDD where *R_in_* = 33 Ω, *C_out_* = 1 nF, *R_out_* = 2200 Ω and the Schottky diode is the model HSMS-2850 [42] by Broadcom. The SDD is etched on the commercial Rogers AD1000 dielectric substrate. The SDD is based on a series configuration, essentially working as a half-wave rectifier. The working principle is the envelope detection of the incoming signal produced by the signal rectification [43]. The combination of *C_out_* and *R_out_* in parallel at the output forms a RC low-pass filter which rejects the high-frequency components. The main problem of the proposed configuration is the difficulty to match the input over a broad bandwidth. For this reason, the designed input matching network is based on a resistive model. This scheme gives a good matching within the whole required bandwidth, as it can be seen in Figure 17a. Figure 17b shows the detected voltage versus input power for the limiting and central frequencies of the interrogation bandwidth (1.6, 1.75 and 1.9 GHz). The detected voltage varies between 9.44 mV and 0.84 V for an input power between −15 dBm and 10 dBm respectively. The received power can be computed as:

(4) $$Received\;Power=VCO\;Power\;\left[\mathrm{dBm}\right]+Broadband\;Amplifier\;Gain\;\left[\mathrm{dB}\right]+\left|S_{21rad}\right|\;\left[\mathrm{dB}\right]$$

resulting in values within the range of the SDD input power.

### 2.4. IoT Mini-Computer Layer

The IoT mini-computer used is the Arduino model MKR1000 [31]. This mini-computer has 28 pins, but only four are needed: *VIN* for the board power supply, *GND* connected to common ground of the reader, *A1* which is proportional to the output of the RF interface (*val* signal) and *A2* which is proportional to the periodic triangular sweep function (*sync* signal). *A1* and *A2* are the analog inputs #1 and #2 of the Arduino MKR1000 mini-computer. Both analog inputs have ADCs to digitize the input signals. The ADCs are set to work with 10 bits resolution. The sampling rate is 1/(120 μs) approximately, which is much higher than the sampling rate needed for sampling the detected signal from the sensor and the triangular sweep function.

**Figure 18:**
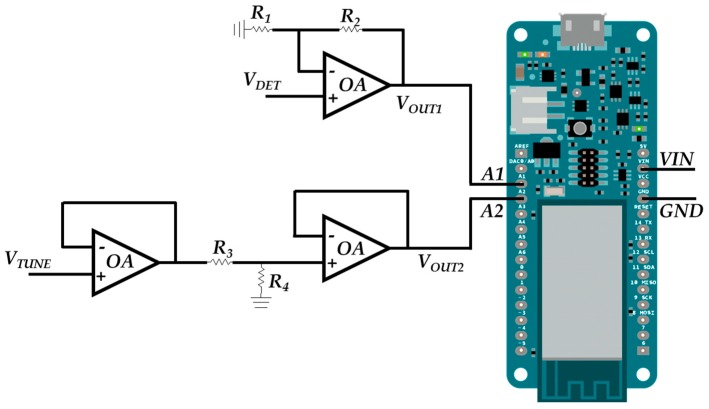
Pinout and signal conditioning circuits for the Arduino MKR1000 board.

Some operational amplifiers (OA) based circuits are used as signal conditioning before the analog inputs of the mini-computer. Figure 18 shows the used pinout and signal conditioning circuits. The maximum received input power of the SDD is 10 dBm (explained in the previous section), which corresponds to a detected voltage *V_DET_* equal to 840 mV (Section 2.3). Thus, the objective of the non-inverter amplifier configuration connected to the *A1* pin is to amplify the output of the RF interface voltage up to 3.3 V, which is the maximum Arduino input voltage. This improves the notch detection by using the whole dynamic range of the Arduino ADCs used for the data acquisition. For this reason, the gain factor 1 + *R*_2_*/R*_1_ of non-inverter amplifier is set to 3.55. On the other hand, the purpose of the signal connected to the pin *A2* is the digitization of the periodic triangular signal. For this reason, it is necessary to attenuate the signal *V_TUNE_* with a highest voltage at 15.5 V below 3.3 V, which is the maximum Arduino input voltage. In order to obtain this attenuation, two OA-based stages are used in a buffered voltage divider configuration. A voltage divider is placed between two voltage followers in order to eliminate the loading effects. The voltage-divider factor *R*_4_*/(R*_4_ + *R*_3_*)* must set below 0.21 to achieve an attenuated triangular signal with a maximum voltage of 3.3 V at the analog pin *A2*. The conditioning circuits are implemented by using standard resistors and the LM324N integrated circuit [44], which contains the OAs.

**Figure 19:**
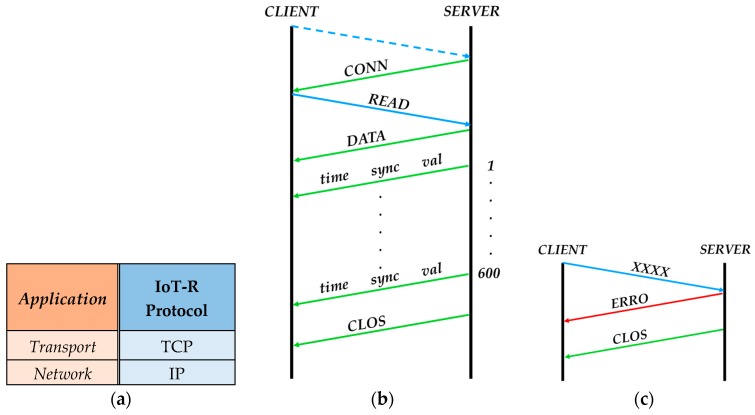
Protocol stack model (**a**), communication protocol in a normal situation (**b**) and in a situation where an incorrect command is sent by the *CLIENT* (**c**).

Finally, the digitized signals can be remotely monitored through the Internet thanks to the IoT functionalities of the mini-computer. The IoT-R is connected to the Internet through the WiFi interface of the Arduino board. The sensing data can be remotely obtained following the communications protocol shown in Figure 19. In Figure 19a the protocol stack model is shown. The proposed IoT-R protocol is defined as an user command protocol. The communication protocol is mounted over TCP/IP in the application layer. On the other hand, with this scheme the reader has an IP address associated. Moreover, the reader is listening the requests associated to this protocol at port 23 of TCP. The data acquisition through the Internet, between a *SERVER* and a *CLIENT*, is possible thanks to this protocol. In this protocol, the *SERVER* is the IoT-R and the *CLIENT* is the remote device asking for the sensing data. The protocol is based on four letter commands where the communication (Figure 19b) is as follows: first of all, the *SERVER* is waiting for incomming connections; then, the connection between the *CLIENT* and the *SERVER* is established and the *SERVER* sends the command *CONN* to the *CLIENT* confirming the connection; once the connection is confirmed, the *CLIENT* sends the command *READ* which means a request for reading the sensor; after that, the *SERVER* sends the command *DATA* which means that it will start sampling the sensor response and sending the data packets; finally, after sending all the packets, the *SERVER* sends the command *CLOS* which means the correct ending of the communication. The data packets are divided into three values: *time* (clock time in ms for each sample), *sync* (a sample of the digitized triangular signal) and *val* (a sample of the digitized response of the sensor). 600 samples are sent to the *CLIENT* with the current configuration, which results in six signal periods. On the other hand, if the *CLIENT* sends an incorrect command (*XXXX* in Figure 19c) the *SERVER* answers with the command *ERRO* which means that the command is unknown. Then, the *SERVER* sends the command *CLOS* ending the communication.

**Figure 20:**
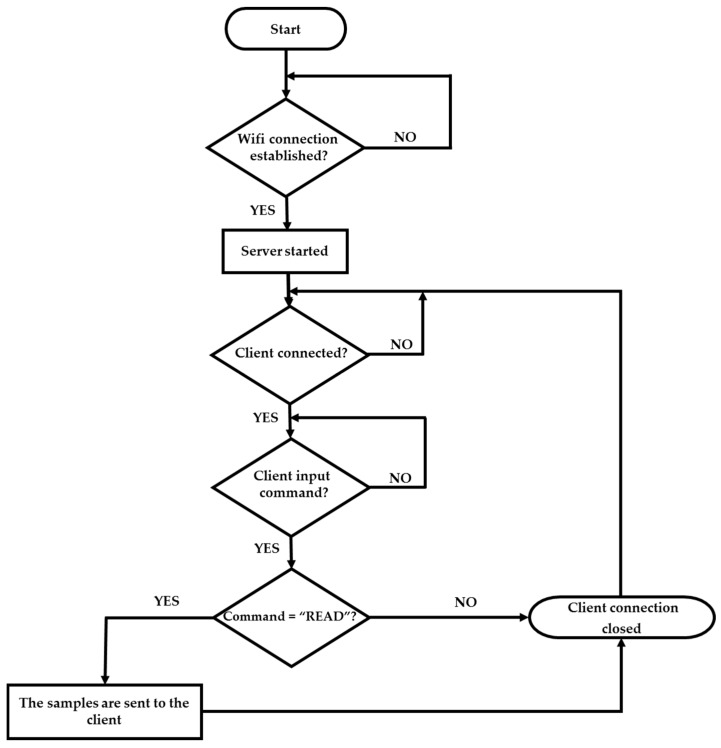
Flow chart of the IoT reader (*SERVER*) program.

The flow chart of the IoT-R *SERVER* program is shown in Figure 20. The program works as follows: after starting the program, it waits until the reader is linked to the WiFi network and the *SERVER* is started. Then, the *SERVER* waits until a *CLIENT* is connected. After the connection is established, the *SERVER* waits for an input command. Once the *SERVER* receives the input command *READ* from the *CLIENT*, the samples are sent and the *CLIENT* connection is finished, otherwise the *CLIENT* connection is closed without sending the samples. Finally, after closing the *CLIENT* connection the *SERVER* waits until other *CLIENT* establishes a connection for starting the process again.

## 3. Results

**Figure 21:**
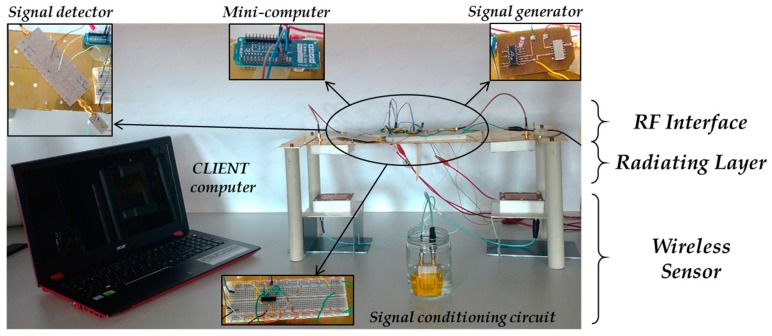
Demonstrator containing all the system elements.

After testing the different reader layers separately, the whole IoT-R is assembled. The experimental setup of the IoT-R system is shown in Figure 21 including the radiating layer, the RF interface, the mini-computer, the *CLIENT* computer and the wireless sensor. The different active layers are biased by the power unit. The used power unit is based on LM350AT regulators [45]. Finally, the IoT-R is connected to the Internet through the WiFi interface, as explained in Section 2.4. In order to test the validity of the whole system for the proposed liquid sensing application, the submersible sensor is immersed in different LUTs. Table 2 shows the measured samples as well as their relative permittivities. It can be noticed that the relative permittivity of the selected samples is between 1 and 6. The measured results are remotely retrieved from the Internet by running a *CLIENT* in a laptop. This *CLIENT* is programmed in Matlab. A different execution of the *CLIENT* program is run for each sensor immersion.

**Figure 22:**
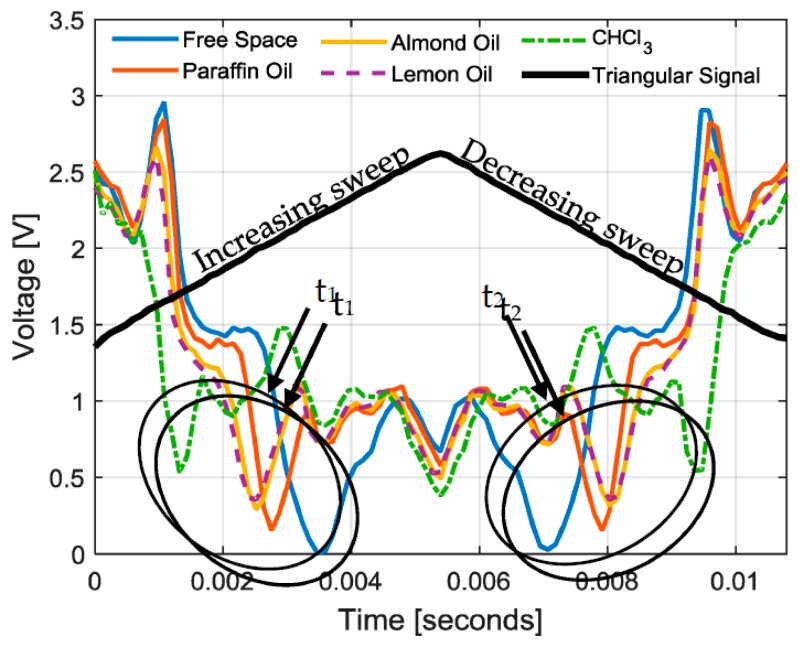
Measurements of the whole system (signals received by the *CLIENT*).

Figure 22 shows the measurements of the whole sensing system for the different samples. A single period of the data received by the *CLIENT* for each sample is represented. The triangular signal (*sync*) is similar for all the samples and is proportional to the signal which controls the frequency sweep (*V_TUNE_*). In particular, two frequency sweeps are performed per period: the first one from 1.6 to 1.9 GHz (increasing sweep) and another one from 1.9 to 1.6 GHz (decreasing sweep). In each sweep, a minimum voltage occurs at a different time for each measurement: *t*_1_ in the increasing sweep, and *t*_2_ in the decreasing sweep. These minima are related to the resonant frequency of the sensor for different LUTs, as explained in Section 2.3. Thus, the detection of the whole sensing system relies in the time difference between *t*_2_ and *t*_1_ (Δ*t* = *t*_2_ − *t*_1_). In Table 2, the time differece, Δ*t*, related to each sample is included. It can be seen a time difference between 3.59 ms and 8.04 ms for values of relative permattivity between 1 and 5.77, corresponding to free space and CHCl_3_ respectively. Each measured sample presents a different Δ*t*. Differences are detected even in the case of almond and lemon oils, whose relative permittivities are close.

**Figure 23:**
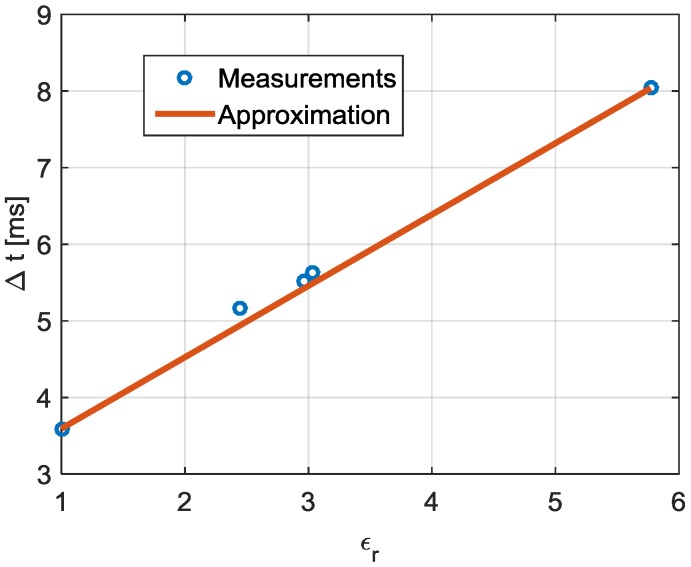
Time difference (Δ*t*) of the measured liquids (blue dots) and the linear approximation (solid red).

Figure 23 shows the dependency of the relative permittivity with the time difference (Δ*t*) for each sample. The measured results show a linear relation between the time difference and the relative permittivity of each sample. For this reason, the linear approximation of Equation (5) is also included. This approximation can be used to easily estimate the relative permittivity of the sample, *ε_r_*, after the measurement of the time difference between the two minima detected in the periodic response signal. The estimation of *ε_r_* can be done by using the Equation (6). The resulting mean error between the measurements and the fitted curve is 1.71%.

(5) $$\Delta t\left[\text{ms}\right]=0.93\cdot \varepsilon_{r}+2.66$$

(6) $$\varepsilon_{r}=1.07\cdot \Delta t\left[\text{ms}\right]-2.86$$

## 4. Conclusions

A novel IoT-R for passive wireless electromagnetic sensors has been proposed. It is based on a layer model formed by: the radiating layer, the RF interface, the IoT mini-computer and the power unit. This work is focused on the implementation of a demonstrator of the whole sensing system. For this purpose, a design of all the layers has been done. These layers have been fully described and independently measured before assembling together to build the whole IoT-R. On the other hand, the data acquisition through the Internet is possible thanks to the communication protocol proposed.

The IoT-R has been tested with a submersible sensor for permittivity characterization of liquids [30]. The sensor used in [30] represents a wired solution, while the IoT-R proposed in this work is capable of detecting the backscattered signal from the sensor leading to a wireless solution. Once manufactured and measured each layer, the sensor has been interrogated remotely through the Internet by a *CLIENT* running in a laptop. This used protocol is mounted over TCP/IP in the application layer. The IoT-R has an IP address associated while it is listening the requests at port 23 of TCP.

Finally, in order to show the validity of the whole system the IoT-R has been tested with different liquids. The results show that the system can estimate the permittivity of liquids. These results can be approximated to a linear regression, which present a small error with respect to the measurements. One of the most interesting application of the proposed system is the possibility of doing remote measurements of harmful substances avoiding the direct contact with them.

The proposed scheme represents an important novelty because, up to the authors’ knowledge, it is the first wireless reader of passive electromagnetic sensors including IoT functionalities. Moreover, the proposed reader is a low-cost and portable solution. It is remarkable that the IoT-R can be used with other wireless electromagnetic sensors, which transduce the magnitude variations in the frequency domain. Finally, the authors are working on a further integration of the radiating layer and the sensors with the antennas. The results will be shown in future works.

## Figures and Tables

**Table 1:** Measured resonant frequency of the radiating layer transmission coefficient ($\;{\left|{S_{21}}_{rad}\right|}^{2}$) for different LUTs.

Material	Relative Permittivity (*ε_r_*)	Resonant Frequency (*f_r_* [GHz])
Free Space	1	1.804
Paraffin Oil	2.45	1.748
Almond Oil	2.96	1.739
Lemon Oil	3.03	1.737
CHCl_3_	5.77	1.649

**Table 2:** Results obtained with the whole sensing system.

Material	Relative Permittivity (*ε_r_*)	*t*_1_ [ms]	*t*_2_ [ms]	Δ*t* (*t*_1_ − *t*_2_) [ms]
Free Space	1	3.48	7.07	3.59
Paraffin Oil	2.45	2.76	7.91	5.16
Almond Oil	2.96	2.52	8.03	5.51
Lemon Oil	3.03	2.40	8.03	5.63
CHCl_3_	5.77	1.32	9.36	8.04
